# Omacetaxine Mepesuccinate: A New Treatment Option for Patients With Chronic Myelogenous Leukemia

**Published:** 2013-07-01

**Authors:** Sandra E. Kurtin, Lisa Matta

**Affiliations:** From University of Arizona Cancer Center, Tucson, Arizona

The discovery of the BCR-ABL oncogene and the subsequent development of agents targeting this oncogene revolutionized the treatment of chronic myelogenous leukemia (CML). The BCR-ABL fusion gene, located on the Philadelphia (Ph) chromosome, encodes an oncoprotein with constitutively active Abl kinase activity. This activity is thought to promote disordered myelopoiesis with accelerated cell growth, decreased apoptosis, weakening of cellular adhesion, and ultimately transformation of normal myeloid cells into the abnormal cells characteristic of CML (Druker et al., 1996; Faderl et al., 1999).

An estimated 5,920 new cases of CML and an estimated 610 deaths are predicted for 2013 (American Cancer Society [ACS], 2013). Although the incidence of CML has remained fairly stable over the past 15 years (4,300 new cases in 1997), death rates have decreased substantially (2,400 deaths in 1997; ACS, 2013). This reduction in mortality is attributed to the development of tyrosine kinase inhibitor (TKI) therapy (ACS, 2013). Thus, oral TKIs are preferred first-line agents for treatment of chronic-phase CML (CML-CP) with the goal of maintaining the chronic phase of the disease (Figure 1). There are currently five oral TKIs approved for the treatment of CML: imatinib (Gleevec), nilotinib (Tasigna), dasatinib (Sprycel), bosutinib (Bosulif), and ponatinib (Iclusig). However, not all patients with CML achieve effective control of their disease with TKIs, and some patients do not tolerate TKI therapy. These patients are at increased risk of progression to the more aggressive accelerated phase (AP) or blast phase (BP) of the disease (Radich, 2010).

**Figure 1:**
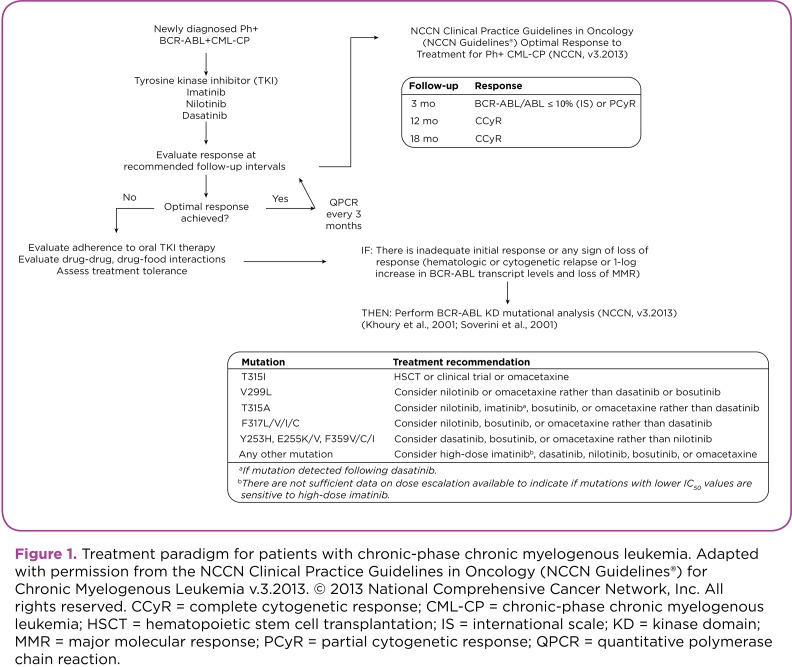
Figure 1. Treatment paradigm for patients with chronic-phase chronic myelogenous leukemia. Adapted with permission from the NCCN Clinical Practice Guidelines in Oncology (NCCN Guidelines®) for Chronic Myelogenous Leukemia v.3.2013. © 2013 National Comprehensive Cancer Network, Inc. All rights reserved. CCyR = complete cytogenetic response; CML-CP = chronic-phase chronic myelogenous leukemia; HSCT = hematopoietic stem cell transplantation; IS = international scale; KD = kinase domain; MMR = major molecular response; PCyR = partial cytogenetic response; QPCR = quantitative polymerase chain reaction.

On October 26, 2012, the US Food and Drug Administration (FDA) approved omacetaxine mepesuccinate (Synribo) for the treatment of adult patients with chronic- or accelerated-phase CML with resistance and/or intolerance to two or more TKIs. The mechanism of action, clinical trial data, clinical management strategies, and response evaluation for patients with CML receiving subcutaneous omacetaxine will be discussed in this article.

## Mechanism of Action

The activity of omacetaxine is independent of targeting the BCR-ABL oncogene. Omacetaxine is a reversible protein translation inhibitor first noted to have activity against CML in preclinical trials conducted in the 1990s (Kantarjian & Cortes, 2011; Visani et al., 1997). Subsequent studies have elucidated several potential mechanisms key to the anti-CML effect of omacetaxine: (1) down-regulation of short-lived proteins, such as the myeloid cell leukemia-1 (MCL-1) protein, that regulate proliferation and cell growth; (2) a reduction in the expression of BCR-ABL thought to be due in part to effects on Hsp90; and (3) apoptotic effects on leukemic stem cells (Chen, Peng, Sullivan, Li, & Li, 2010; Klag et al., 2012; Tang et al., 2006).

## Clinical Trials

Omacetaxine 2.5 mg/m^2^/day administered by continuous infusion for 5 to 14 days together with cytarabine was shown to have clinical activity in early trials in patients with CML (Kantarjian et al., 1999; O’Brien et al., 1995; Stone et al., 2009). However, there was no substantial improvement in complete cytogenetic response (CCyR) rates compared to studies reported for single-agent imatinib, the standard of care at that time. Subsequently, pharmacokinetic studies showed similar properties of omacetaxine when given by subcutaneous (SC) injection (Levy et al., 2006).

Cortes and colleagues recently reported the results of a phase II prospective, multicenter, single-arm study evaluating SC omacetaxine in 62 patients with T315I-mutated CML-CP resistant to one or more TKI therapies, including imatinib (Cortes et al., 2012). Patients received SC omacetaxine 1.25 mg/m^2^ twice daily for 14 consecutive days every 28 days until hematologic response or a maximum of six cycles, followed by a maintenance regimen of omacetaxine 1.25 mg/m^2^ on days 1 through 7 every 28 days (see Table). Patients received a median of 7 cycles (range: 1–41 cycles); 48 (77%) patients achieved or maintained a complete hematologic response. Median response duration was 9.1 months, and 14 (23%) patients achieved a major cytogenetic response (MCyR), including CCyR in 10 (16%) patients. Median progression-free survival was 7.7 months.

**Table 1:**
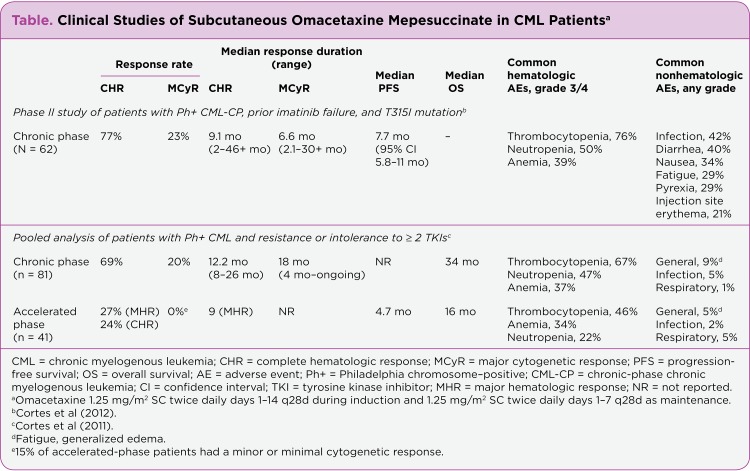
Table 1. Clinical Studies of Subcutaneous Omacetaxine Mepesuccinate in CML Patients

During this phase II study, grade 3/4 hematologic adverse events were common (thrombocytopenia, 76%; neutropenia, 44%; and anemia, 39%) but effectively managed with dose delays and supportive care. Nonhematologic adverse events were mostly grade 1/2 and included infection (42%), diarrhea (40%), and nausea (34%).

A combined analysis of the parallel studies CML 202 (enrolled September 2006 through March 2010) and CML 203 (enrolled March 2007 through June 2009) substantiate these data (Table). A recent ad hoc analysis of these pooled data from 203 patients describes 11 patients (9 CML-CP, 2 CML-AP) who, after a median follow-up of 38.8 months, continue on treatment as of March 2012. Seven of the nine patients with CML-CP achieved MCyR, one patient achieved a partial cytogenetic response, and one patient did not achieve a cytogenetic response (Kantarjian et al., 2012). No patient with CML-AP achieved a MCyR (Kantarjian et al., 2012). The number of cycles was 35 for patients with CML-CP (range: 26–53 cycles) and 20.5 for the two patients with CML-AP (range: 19–22). Treatment-emergent adverse events were similar to those reported in the phase II studies.

## Clinical Management of Adverse Events

**Figure 2:**
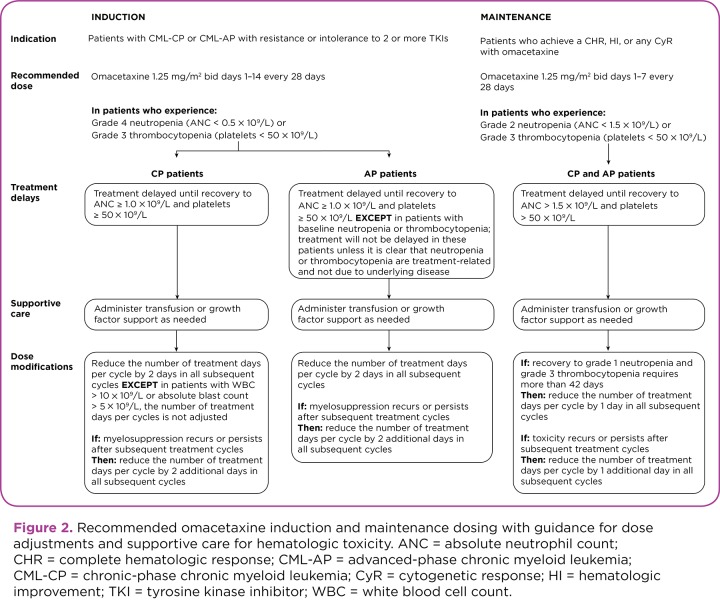
Figure 2. Recommended omacetaxine induction and maintenance dosing with guidance for dose adjustments and supportive care for hematologic toxicity. ANC = absolute neutrophil count; CHR = complete hematologic response; CML-AP = advanced-phase chronic myeloid leukemia; CML-CP = chronic-phase chronic myeloid leukemia; CyR = cytogenetic response; HI = hematologic improvement; TKI = tyrosine kinase inhibitor; WBC = white blood cell count.

Myelosuppression is common in patients receiving omacetaxine for the treatment of CML. Monitoring should include a complete blood cell count, differential, and platelet count weekly during the induction and initial maintenance cycles to establish a trend and allow early identification of cytopenias. Dose delays are recommended based on the severity of the cytopenias (Figure 2). The desired goal for all dose adjustments during maintenance therapy is to establish and maintain as close to a 28-day dosing cycle as possible. Maintenance therapy should continue as long as the patient is clinically benefiting from treatment with an acceptable level of toxicity. In the phase II trial by Cortes and colleagues, supportive care included filgrastim in 13% of patients and epoetin/darbepoetin in 21% of patients (Cortes et al., 2012).

Injection site reactions occurred in 21% of patients and were generally mild and transient. Techniques to reduce injection site reactions associated with chemotherapy administration were discussed in a recent issue of this journal (Kurtin, Knopf, & Milliron, 2012). Mild diarrhea (grade 1) occurred in 33% of induction cycles, requiring no dose delays. Treatment-related nausea may be effectively managed with available antiemetic agents. Hyperglycemia (grade 1/2) was observed in nondiabetic patients, thus diabetic patients receiving omacetaxine should be monitored more closely for hyperglycemia (Legros et al., 2007).

## Evaluation of Treatment Response

Treatment response in CML is defined by achievement of key hematologic, cytogenetic, and molecular milestones at defined intervals (Kantarjian & Cortes, 2011; NCCN, 2013; see Figure 1). Achievement of a CCyR, defined as the absence of the Ph+ clone by metaphase cytogenetics, remains the most important initial goal of therapy (NCCN, 2013). In stable CCyR (usually after 2 years of therapy), sudden blastic transformations are rare (Cortes, Quintas-Cardama, & Kantarjian, 2011). Achievement of a major molecular response (MMR), defined as a 3-log reduction in the BCR-ABL transcripts measured by real-time quantitative polymerase chain reaction (RQ-PCR), also has been shown to correlate with improved progression-free survival (Cortes, Quintas-Cardama, & Kantarjian, 2011. More recently, the international scale (IS) was created to standardize RQ-PCR testing for BCR-ABL across different laboratories, with MMR defined as 0.1% on the IS (Hughes et al., 2006). Failure to achieve an optimal response at target follow-up times signals an increased risk of progression to the more aggressive phases of the disease.

Resistance to and intolerance of TKIs are the primary causes of suboptimal treatment response. Primary hematologic resistance in newly diagnosed patients with CML is rare (Radich, 2010). Cytogenetic resistance is observed in approximately 15% to 25% of patients with CML (Radich, 2010). Some patients may have primary resistance or develop secondary resistance due to mutation in the kinase domain (KD) of the BCR-ABL gene, thought to be a result of the inherent genetic instability of the BCR-ABL oncogene over time (Radich, 2010). The T315I point mutation in the KD of BCR-ABL is associated with inferior survival, due largely to primary resistance to most currently approved TKIs (Hughes et al., 2009; Muller et al., 2009). The availability of the novel agent omacetaxine, which has a distinctly different mechanism of action and proven activity in patients with resistance or intolerance to two or more TKIs, including those harboring the T315I mutation, provides an important treatment option for patients with CML (Figure 1).

Survival for CML-CP patients with the T315I point mutation treated with TKIs is estimated to be reduced by 50% compared with patients without the T315I mutation (median survival of 22 months vs. more than 10 years for TKI-responsive CML patients; Nicolini et al., 2009). Additionally, omacetaxine has been shown to potentiate the effects of imatinib and nilotinib by overcoming cytokine rescue of BCR-ABL–positive leukemic cells thought to be mediated primarily by interleukin-3 (Allan, Holyoake, Craig, & Jorgensen, 2011; Klag et al., 2012), offering promising new options for combination therapies.

## Conclusions

Omacetaxine offers an important treatment option for CML patients with resistance or intolerance to TKIs. Administration requires regular monitoring for hematologic toxicities, which can be effectively managed using the recommended dose delays together with supportive care measures. Nonhematologic toxicities are generally mild and manageable. Unlike the TKIs, which are self-administered oral agents, omacetaxine requires SC administration in the clinical setting, offering the opportunity for more frequent contact and monitoring by oncology advanced practitioners. Familiarity with the novel mechanism of action, dosing and administration guidelines, and recommendations for management of adverse events will give the advanced practitioner the tools to provide safe and effective treatment of patients receiving omacetaxine.

## Acknowledgments

Financial support for editorial assistance and figure/table development was provided by Teva Pharmaceuticals, Inc. The authors thank Powered 4 Significance LLC for this editorial support.
